# Determination of *in vitro* antioxidant activity and *in vivo* antineoplastic effects against Ehrlich ascites carcinoma of methanolic extract of *Sphagneticola calendulacea* (L.) Pruski

**DOI:** 10.1016/j.heliyon.2021.e07228

**Published:** 2021-06-09

**Authors:** Md. Wasim Bari, Ariful Islam, Md. Monirul Islam, Mst Julia Sultana, Rashida Afroz, Md. Mahbubur Rahman Khan, Salina Shaheen Parul, A.M. Swaraz, Mohammad Ismail Hossain, Mohammad Amirul Islam

**Affiliations:** a Department of Biochemistry and Molecular Biology, University of Rajshahi, Rajshahi, 6205, Bangladesh; b Department of Cellular and Molecular Anatomy, Hamamatsu University School of Medicine, Hamamatsu, Shizuoka 431-3192, Japan; c Department of Biochemistry, Hamamatsu University School of Medicine, Hamamatsu, Shizuoka 431-3192, Japan; d Department of Neurophysiology, Hamamatsu University School of Medicine, Hamamatsu, Shizuoka 431-3192, Japan; e Department of Biochemistry, Sylhet MAG Osmani Medical College, Sylhet, Bangladesh; f Department of Genetic Engineering and Biotechnology, Jashore University of Science and Technology, Jashore 7408, Bangladesh

**Keywords:** *Sphagneticola calendulacea*, Antioxidant activity, Antineoplastic activity, Ehrlich ascites carcinoma, Apoptosis

## Abstract

**Background and aim:**

*Sphagneticola calendulacea* (L.) Pruski *(S. calendulacea)* is a well-known medicinal plant that is widely used in traditional medicine. This study was conducted to evaluate the antioxidant and antineoplastic properties of *S. calendulacea* leaf.

**Experimental procedure:**

Methanol was used as a solvent to prepare leaf extract from the dried leaf of *S. calendulacea* (MESL). We have evaluated the total phenolic and flavonoid contents of MESL, and using five different assays; we have also evaluated the antioxidant property of MESL. *In vivo* antineoplastic activity of MESL against Ehrlich ascites carcinoma (EAC) cells in experimental mice was also explored.

**Results:**

The phytochemical analysis of MESL exhibited the total phenolic, and total flavonoid contents in the dry extracts were 32.86 mg/gm of gallic acid equivalent and 127.26 mg/gm of catechin equivalent, respectively. Moreover, MESL also showed promising scavenging activity in all assays (IC_50_ value ≥70.5 μg/mL). In the *in vivo* antineoplastic assay, MESL showed increased EAC cell death at the doses of 50 mg/kg and 100 mg/kg body weight. MESL administration also induced apoptosis of EAC cells, significantly inhibited EAC cell growth, and increased the life span of EAC cell-bearing mice compared to EAC cell-bearing control mice.

**Conclusions:**

All findings of this study suggest potential antioxidant and antineoplastic properties of MESL.

## 1 Introduction

Since ancient time, medicinal plants and their extracts are being used to heal many infectious diseases and contributes as major sources of therapeutic agents. Medicinal plants possess many therapeutic potentialities due to their phytoconstituents and antioxidant property in various chronic diseases [1, 2]. According to the World Health Organization, it was found that approximately 80% of modern civilizations rely on the substance of plants. Herbs generate bioactive compounds, including antioxidants that react to free radicals and suppress microbial growth in other species of the environment [3]. Medicinal plants play a leading role in resisting or regulating diseases due to their constituents of antioxidant properties usually associated with various polyphenol compounds [4]. Naturally antioxidant products such as phenolics and flavonoids are predominantly found in edible and inedible plants that exhibit antioxidants, antimicrobial, anti-carcinogenicity, and other biological activities [5, 6, 7].

Cancer is one of the leading causes of death all over the world. The number of new cases rises globally and is expected to increase by 70% within 2040 [8]. Carcinogenesis arises due to the mutation in deoxyribonucleic acid (DNA) by increased reactive oxygen species (ROS) and reactive nitrogen species (RNS) produced during oxidative stress [9]. ROS and RNS production rate is increased day by day due to prolonged exposure to environmental and biological factors such as climatic alteration, transportation, ultraviolet and ionizing radiations, heavy metals, and alteration in food habits [10, 11]. The compounds of plant origin can essentially remove the free radicals that mediate toxicity, and for a wide variety of herbs with numerous compounds with these activities have been recorded already [12, 13]. Researchers are particularly interested in natural antioxidants due to their relatively low adverse effects, especially plant ones.

*Sphagneticola calendulacea* (L.) Pruski (*S. calendulacea*) is a small branched herb locally known as Bhimra or Bhringraj, rich in polyphenols [14, 15]. Traditionally plant leaves are used in herbal medicine due to possessing cough-relieving, antipyretic, detoxification, antiphlogistic, anti-microbial, anti-inflammatory, anticancer, antidiabetic, and CNS-depressant activities [14, 15, 16, 17, 18]. Previous studies suggest that *S. calendulacea* is rich in polyphenols and a major source of antioxidants which might have anticancer property [12, 15, 19]. The above overall information from the literature indicates that *S. calendulacea* leaf may have antioxidant and anticancer activities. As far we know, the determination of quantitative polyphenols, antioxidant and anticancer activity of *S. calendulacea* leaf against Ehrlich ascites carcinoma (EAC) cells were not studied yet. The present study implies evaluating polyphenol contents, antioxidant capacity, and anticancer activity of methanolic extract of *S. calendulacea* leaf (MESL) against EAC cell.

## 2 Materials and methods

### 2.1 Chemicals and reagents

Folin-ciocalteu reagent (FCR), sodium carbonate (Na_2_CO_3_), aluminum chloride (AlCl_3_), catechin, potassium ferricyanide [K_3_Fe(CN)_6_], trichloroacetic acid (TCA), ferric chloride (FeCl_3_), copper chloride (CuCl_2_), neocuproine, 2, 2′-diphenyl-1-picryl-hydrazyl (DPPH•), 2, 2′-azino-bis(ethylbenzthiazoline-6-sulfonic acid) diammonium salt (ABTS•), and ascorbic acid were purchased from Sigma-Aldrich (St. Louis, MO, USA). Potassium persulfate (K_2_S_2_O_7_), sodium nitroprusside, N-(1-naphthyl) ethylenediamine dihydrochloride (NED), sulfanilic acid, glacial acetic acid (Karlsruhe, Germany), and gallic acid were purchased from (Wako pure chemicals Ltd, Japan). Methanol and chloroform were purchased from E-Mark (Germany). All the chemicals used in this study were of analytical grade.

### 2.2 Collection of plants materials and extractions

The whole plant of *Sphagneticola calendulacea* (L.) Pruski was collected from Rajshahi University Campus, Rajshahi-6205, Bangladesh, in November 2020. Collected plant materials were identified with the help of standard flora Ahmed *et al*., (2008) [20] by the renowned taxonomist Dr. A.H.M. Mahbubur Rahman, Professor, Department of Botany, University of Rajshahi, Bangladesh. A voucher specimen (Herbarium Accession Number 389) has been kept in the herbarium library of the Department of Botany, University of Rajshahi, Bangladesh, for future reference. Leaves were washed with water to remove mud and then shade dried at 25–28 °C for seven days. After shade drying, the plant materials were ground to a coarse powder by a grinding machine (FFC-15, China) and stored in an airtight container at room temperature for further use. About 120 gm of dried powder of *S. calendulacea* leaves was placed in a clean round-bottomed bottle, added 500 mL methanol, and left for seven days with occasional shaking. After that period, the mixture was filtered through Whatman no.1 filter paper to get a pure solution. Then the solvent was evaporated using a rotary evaporator at 39 °C to yield MESL. The concentrated extract was preserved for further experimental use at 4 °C in storage vials and utilized within a month.

### 2.3 Estimation of total phenolic and flavonoid contents

Total phenolic contents of MESL were estimated according to the FCR method with slight modification [21], where gallic acid was used as a standard. 0.3 mL of crude extracts (1 mg/mL) were dissolved in methanol and mixed with 2.5 mL of 10 times diluted FCR using water. Then those mixtures were allowed for 5 min at room temperature and followed by the addition of 2.25 mL of 6% Na_2_CO_3_. The tubes were vortexed for reaction and allowed to stand for 90 min at 25 °C for color development, and the absorbance was measured at 650 nm using a UV-spectrophotometer. Total phenolic contents were calculated from the calibration curve, and results were expressed as mg of gallic acid equivalent (GAE) per gm of dry extract.

Total flavonoid contents of crude extract were determined by the AlCl_3_-colorimetric method with slight modification [22]. 0.5 mL of the extracts (1 mg/mL) were made up to 1 mL with methanol mixed with 2.25 mL of distilled water followed by the addition of 0.15 mL of 5% NaNO_2_ solution and kept at room temperature for 6 min. After that, 0.3 mL of a 10% AlCl_3_.6H_2_O solution was added and allowed to stand for another 5 min before adding 1 mL of 1 M NaOH. The mixture was allowed to stand for 15 min, and the absorbance was measured at 430 nm using a UV-spectrophotometer. The total flavonoid contents were calculated from a calibration curve, and the result was expressed as mg of catechin equivalents (CAE) per gm of dry extract.

### 2.4 Determination of ferric reducing antioxidant power (FRAP)

The ferric reducing antioxidant capacity was evaluated according to the method proposed by Oyaizu [23] with slight modification. Briefly 0.25 mL of extracts were mixed with potassium buffer (0.625 mL, 0.2 M, pH 6.7) and 0.625 mL of 1% potassium ferricyanide [K_3_Fe(CN)_6_]. The mixture was incubated at 50 °C for 20 min. After incubation, 0.625 mL of 10% trichloroacetic acid solution was added to each tube, and the mixture was centrifuged at 3000 rpm for 10 min. Clear supernatant (1.8 mL) was mixed with an equal amount of distilled water and 0.36 mL of FeCl_3_ (0.1% w/v) solution, and then absorbance was recorded at 700 nm. Here ascorbic acid was used as standard.

### 2.5 Copper ion reducing assay (CUPRAC)

The cupric reducing antioxidant activity of the methanol extract was determined by the method described by Çekiç *et al.* [24] with slight modification. Different concentrations of sample solutions (1 mg/mL; 0.5 mL) were added to premixed reaction mixture containing CuCl_2_ (1 mL, 10 mM), neocuproine (1 mL, 7.5 mM) and ammonium acetate (NH_4_Ac) buffer (1 mL, 1 M, pH 7.0). Similarly, a blank was prepared by adding sample solution (0.5 mL) to the premixed reaction mixture (3 mL) without CuCl_2_. The sample and blank absorbance were read at 450 nm after 30 min of incubation at room temperature. Ascorbic acid was used as a standard sample.

### 2.6 DPPH free radical scavenging activity

Free radical scavenging activity of MESL was tested against 2, 2-diphenyl-1-picryl-hydrazyl (Sigma-Aldrich) radical using previously described method [25]. In brief, 1 mL of MESL extract solution in methanol and ascorbic acid were mixed with 3 mL of DPPH solution (0.1 mM) in methanol. The reaction mixture was then left in the dark at room temperature for 30 min. Finally, the absorbance of each extract was measured at 517 nm. The free radical scavenging activity of each sample was calculated by using the following formula:

DPPH Radical scavenging rate (%) = [{A_0_ – A}/ A_0_] × 100

where “A_0_” (control) was the absorbance of DPPH blank solution, and “A” was the final absorbance of the tested sample after 30 min of incubation.

### 2.7 ABTS free radical scavenging assay

Determination of ABTS scavenging capacity was carried out as reported by Re *et al.* [26]. The radical cation was prepared by mixing 7 mM ABTS stock solution with 2.45 mM potassium persulfate (1/1, v/v) and leaving the mixture for 10–16 h in a dark place at room temperature until the reaction was completed. ABTS • + solution was diluted with water to achieve an absorbance of 0.70 ± 0.02 at 734 nm. Then, 1 mL of various sample solution concentrations was mixed with 3.0 mL of ABTS •+ solution and vortexed. After 6 min of incubation, the absorbance was measured at 734 nm. Ascorbic acid was used as the positive control. The scavenging rate was calculated using the formula:

ABTS radical scavenging rate (%) = [{A_0_ – A}/ A_0_] × 100

where “A_0_” (control) was the absorbance of ABTS blank solution, and “A” was the final absorbance of the tested sample after 6 min of incubation.

### 2.8 Nitric oxide scavenging assay

The method of Shukla *et al.* was used to determine the nitric oxide radical scavenging activity of MESL with some modification [27]. A volume of 2 mL of 10 mM sodium nitroprusside was dissolved in a 0.5 mL phosphate buffer saline (pH 7.4) and mixed with 0.5 mL of the sample at various concentrations. The mixture was then incubated at 25 °C. After 150 min of incubation, the incubated mixtures were mixed with 0.5 mL of Griess reagent containing 1% (w/v) sulphanilamide, 2% (v/v) H_3_PO_4_ and 0.1% (w/v) N-(1-naphthyl) ethylenediamine dihydrochloride. The follow-through was then incubated at room temperature for 30 min, and its absorbance was measured at 546 nm. Ascorbic acid was used as a reference standard. The amount of nitric oxide radical inhibition was calculated following this equation:

Percent (%) inhibition of NO radical = [{A_0_ – A}/ A_0_] × 100

where A_0_ is the absorbance of the blank control (NO radical solution without test sample), and A is the absorbance of the test sample.

### 2.9 Animal care

Swiss albino mice (20–25 gm body weight; BW) were collected from the Animal Resources Branch of the International Centre for Diarrheal Diseases and Research, Bangladesh (icddr,b). The mice were kept in polypropylene cages in well-ventilated rooms and maintained under standard laboratory conditions (temperature 25 ± 2 °C; humidity 55 ± 5%) with 12 h dark/light cycle. The mice were allowed free access to standard diet and water and were acclimatized to laboratory conditions for one week before starting the experiment. Ethical permission was approved by the Institutional Animal, Medical Ethics, Bio-Safety and Bio-Security Committee (IAMEBBC), Institute of Biological Sciences, University of Rajshahi, Bangladesh.

### 2.10 Collection of EAC cells

EAC cells were collected from the Indian Institute for Chemical Biology (IICB), Kolkata, India. They were maintained by weekly intraperitoneal inoculation of 10^5^ cells per mouse in the laboratory according to the method described by Habib *et al.* [28].

### 2.11 Studies of EAC cell growth in the animal model

For the therapeutic evaluation of MESL on EAC cell growth, 1.5 × 10^6^ cancer cells were inoculated into four groups of mice on day zero. Treatment was started after 24 h of tumor inoculation. Standard drug bleomycin (Biochem Pharmaceutical, India) at the dose of 0.3 mg/kg body weight (BW), MESL at doses of 50 mg/kg/day BW, and 100 mg/kg/day BW were given by intraperitoneal injection to mice (group 2, 3 and 4, respectively). Group 1 was considered as the untreated control group and given 2% (v/v) dimethylsulfoxide (DMSO). After six days of treatment, animals were sacrificed, and EAC cells were collected by repeated intraperitoneal washing with 0.9% saline. Viable EAC cells were counted with a hemocytometer. The counted viable EAC cells of every mouse of the treated groups were compared with untreated control mice [29].

### 2.12 Studies on survival time and tumor weight

Survival time and tumor weight were determined following the method described by Sur *et al.* [29], with slight modification. Animals were divided into four groups, consisting of 6 mice in each and inoculated with 1.5×10^6^ cells/mouse on day zero. The control group (group 1) was untreated. After 24 h of inoculation, treatment with standard drug bleomycin (0.3 mg/kg BW) and MESL at the doses of 50 and 100 mg/kg BW on groups 2, 3, and 4, respectively. After 15 days of treatment, tumor weight gain in each group was noted. The average BW of each group was also noted on the 15^th^ day after EAC cell inoculation. The survival time was recorded and expressed as mean survival time (MST) in days, and the percent increase of life span (%ILS) was calculated as follows;

% ILS = (MST of treated group / MST of control group) x 100

where MST = (∑ Survival time in days of each mouse in a group)/Total number of mice.

### 2.13 Studies on hematological parameters

To explore the effects of MESL on hematological parameters in EAC cell-bearing mice, 24 mice (Swiss albino mice) were divided into four groups (n = 6) after intraperitoneal injection of EAC cells (1.5 × 10 ^6^ cells/mouse). After 24 h of inoculation, all mice were treated (intraperitoneal injection) with MESL and bleomycin for 14 days as follows;

Group 1 (control): saline (5 mL/kg/mouse/day)
Group 2: MESL treated mice (50 mg/kg/mouse/day).
Group 3: MESL treated mice (100 mg/kg/mouse/day).
Group 4: Bleomycin treated mice (0.3 mg/kg/mouse/day).

Tail vein blood was collected from each mouse on the 15^th^ day after EAC cell inoculation, and hematological parameters such as hemoglobin (Hgb), red blood cell (RBC), and white blood cell(WBC) were measured from each mouse according to the method described by Habib *et al.* [28].

### 2.14 Studies on morphological appearance of EAC cells

Using a fluorescence microscope (Olympus iX71 Korea), the morphological shift in EAC cells in MESL treated and untreated mice was observed. The EAC cells were gathered and dyed for 10 min in the dark and subsequently with Hoechst 33342 at 37 °C, then washed with phosphate buffer saline. Microscopic fluorescence analysis was eventually carried out to observe morphological changes of EAC cells [28].

### 2.15 Statistical analysis

All statistical analyses were performed using IBM SPSS Statics software (version 16). All values were expressed as mean ± standard deviation (SD) and considered statistically significant at *p* < 0.05 (one-way ANOVA followed by Dunnett's test).

## 3 Results

### 3.1 Total phenolic and flavonoid contents

**Figure 1:**
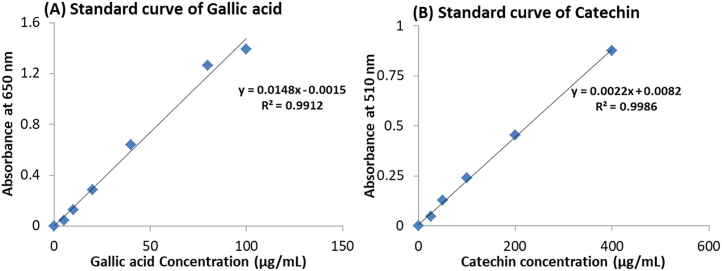
Standard curve of gallic acid (A) and catechin (B) for the determination of total phenolics and total flavonoids, respectively. All data were presented as mean ± SD (n = 3).

To estimate phenolic and flavonoid contents of MESL, calibration curves using different gallic acid and catechin concentrations were made following FCR methods (Figure 1A and B).

Using the calibration curve of gallic acid and catechin, we have estimated total phenolic and flavonoid contents present in MESL, which were shown in Table 1. A higher amount of total phenolic and flavonoid contents were found in MESL in this study.

**Table 1:** Phytochemical contents of MESL.

Name of polyphenols	Contents
Phenolics	32.86 ± 1.18 mg/gm GAE
Flavonoids	127.27 ± 2.77mg/gm CAE

Data were expressed as mean ± SD (n = 3).

### 3.2 Ferric reducing antioxidant power

**Figure 2:**
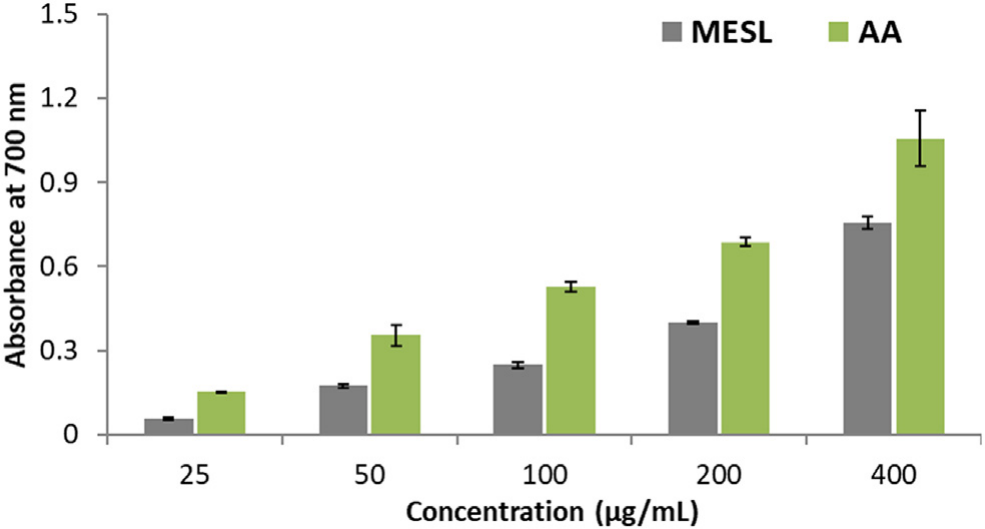
Ferric reducing antioxidant activity of MESL along with the ascorbic acid. All data were presented as mean ± SD (n = 3).

Ferric reducing antioxidant capacity was used to determination of the antioxidant potentiality of MESL. Figure 2 represents the ferric reducing antioxidant capacity of MESL. We have noted gradually increased ferric ion reducing capacity of MESL with increasing concentration. At 400 μg/mL concentration of MESL, we observed the highest amount of reducing capacity (0.06 ± 0.02) compared with ascorbic acid.

### 3.3 Copper reducing antioxidant capacity

**Figure 3:**
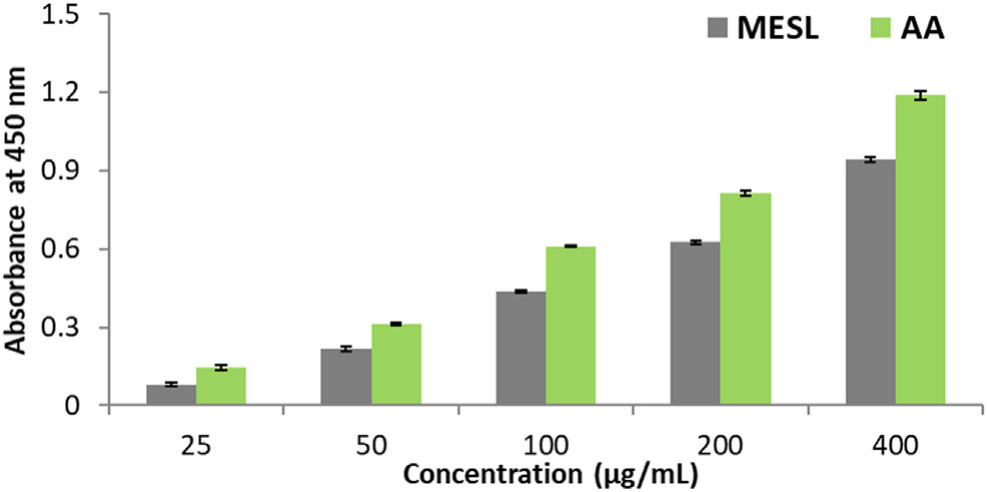
Copper ion reducing activity of MESL along with the standard Ascorbic acid. All values were presented as mean ± SD (n = 3).

In copper ion reducing activity assay, MESL showed gradually increased reducing capacity with increasing concentration (Figure 3). Compared to standard ascorbic acid, MESL showed moderate reducing capacity, and its highest reducing activity was noted at 400 μg/mL concentration.

### 3.4 DPPH free radical scavenging activity

**Figure 4:**
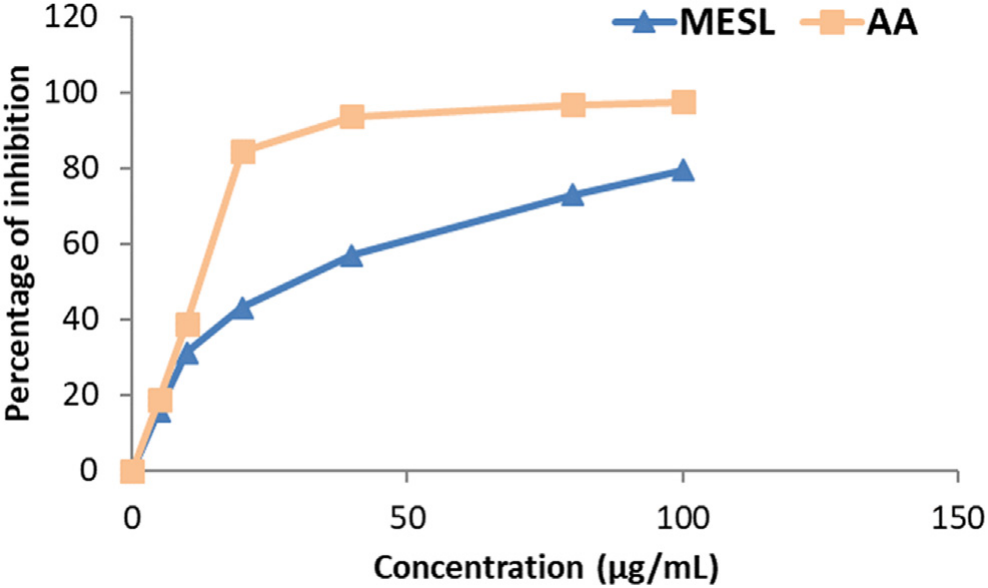
DPPH free radical scavenging activity of MESL and ascorbic acid. All values were expressed as mean ± SD (n = 3).

In this study, the concentration-dependent antiradical activity of MESL against DPPHˉ radical was noted which was presented in Figure 4. Ascorbic acid showed a higher degree of free radical-scavenging activity than that of the plant extract at each concentration point. At the highest concentration of 100 μg/mL, the percentage of scavenging activity of MESL was 79.43, while at the same concentration, the percentage of scavenging activity of ascorbic acid was 97.5. Moreover, the IC_50_ value of MESL was 46.80 μg/mL, while the IC_50_ value for the ascorbic acid was 23.61 μg/mL.

### 3.5 ABTS free radical scavenging activity

**Figure 5:**
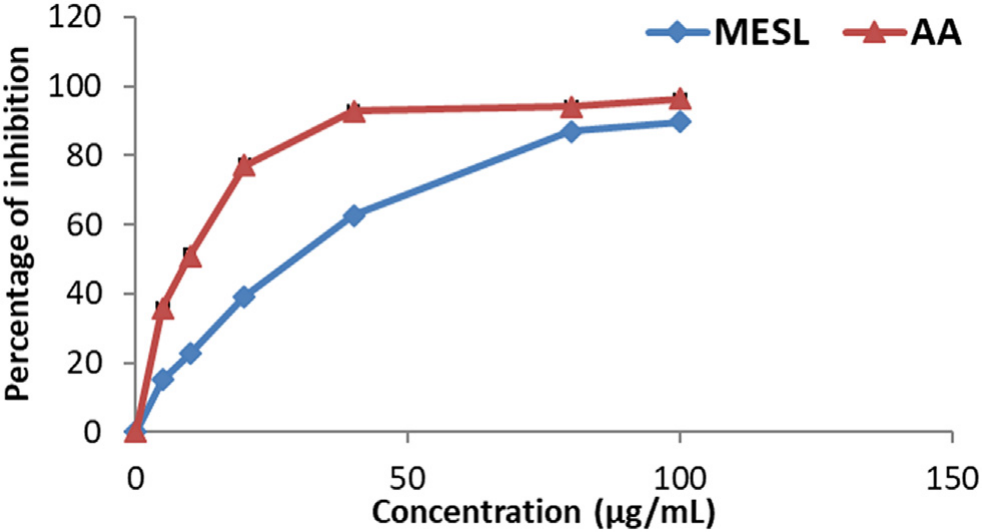
ABTS free radical scavenging activity of MESL and ascorbic acid. All values were expressed as mean ± SD (n = 3).

ABTS• is more reactive than DPPH, and unlike the reactions with DPPH, which involves ‘H^+^’ atom transfer, the reaction with ABTS• involves an electron transfer. In this study, we have also explored the ABTS• radical scavenging capacity of MESL. MESL showed in a concentration-dependent manner. In Figure 5 at the concentration of 100 μg/mL, MEWL showed 89.63% ABTS• radical scavenging capacity, where at the same concentration, ascorbic acid showed 96.41% ABTS• radical scavenging capacity. IC_50_ values for MESL were 35.56 μg/mL, whereas that of ascorbic acid was 17.59 μg/mL.

### 3.6 Nitric oxide scavenging assay

**Figure 6:**
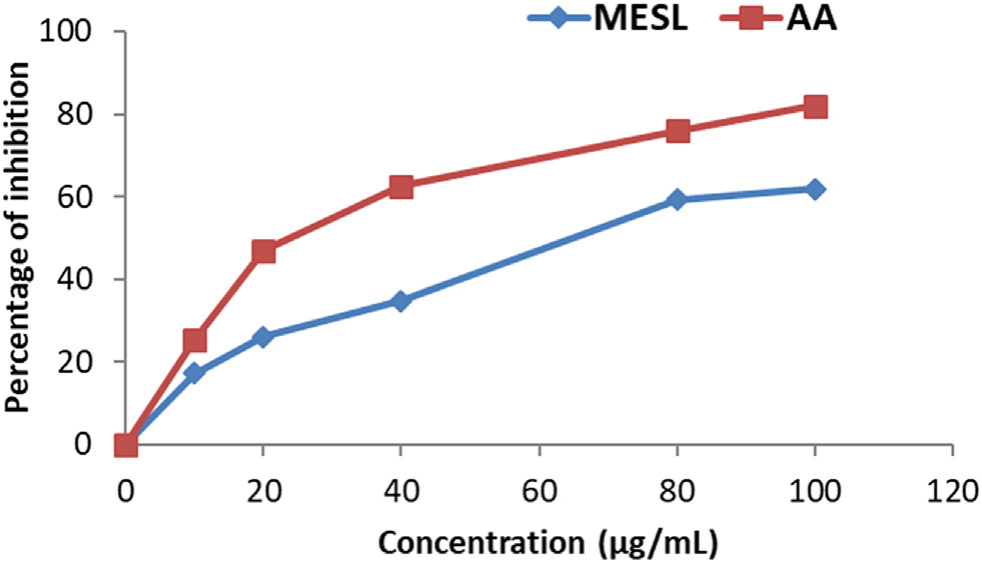
NO free radical scavenging activity of MESL and ascorbic acid. All values were expressed as mean ± SD (n = 3).

Nitric oxide free radical inhibition activity of MESL was shown in Figure 6. MESL exhibited continuously increasing antioxidant activity in the NO radical scavenging assay with increasing concentration. At the concentration of 100 μg/mL MESL, the percentage inhibition of the NO radical was 61.90%, whereas, at the same concentration, ascorbic acid inhibited 82.11% NO radical. The IC_50_ value of MESL was found to 70.50 μg/mL compared to the standard ascorbic acid, whose IC_50_ value was found to 43.38 μg/mL.

### 3.7 Effect of MESL on EAC cell proliferation

**Figure 7:**
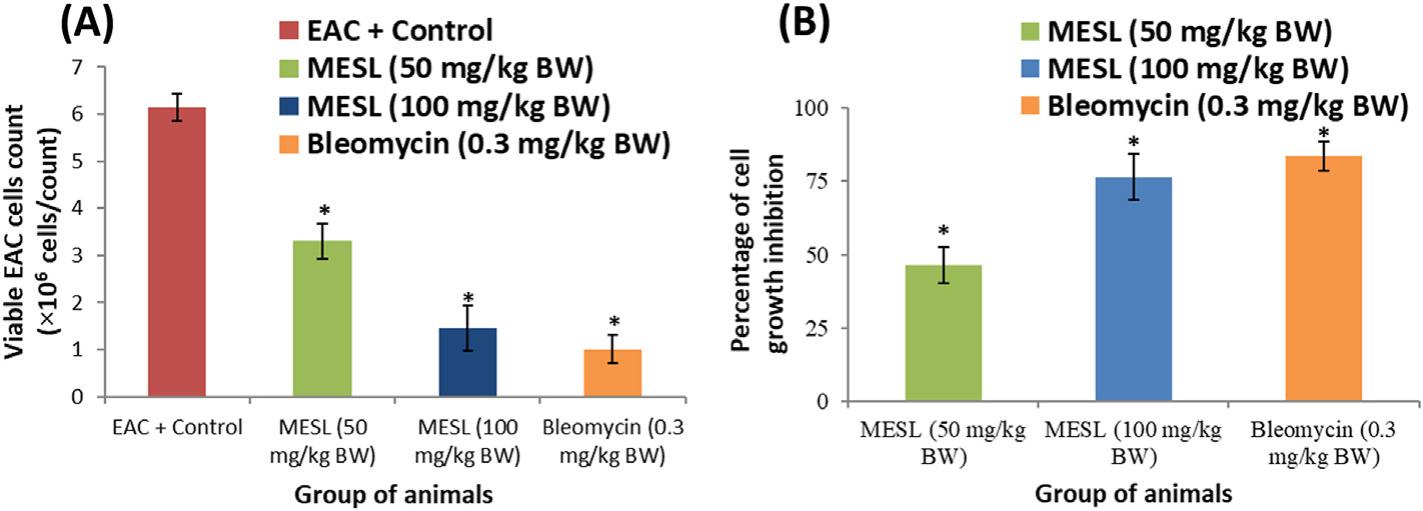
Effect of MESL on viable EAC cells (Figure 7A) and EAC cell growth after seven days of tumor cell inoculation (Figure 7B). All data were expressed as mean ± SD (n = 6). *∗P* < 0.05 compared to EAC control.

*In vivo* antineoplastic activity of MESL against EAC cell-bearing mice was assessed by measuring the parameters such as viable EAC cells, percentage of inhibition in cell growth, and percentage increase in the life span (%ILS). The average number of viable tumor cells per control mouse was (6.15 ± 0.285) x 10^6^ cells/mL. MESL treatment at both doses significantly decreased the number of viable cells (Figure 7A). MESL treatment also inhibited EAC cell growth by 46.33% and 76.41% at the dose of 50 and 100 mg/kg BW, where bleomycin inhibited EAC cell growth by 83.503% at the dose of 0.3 mg/kg BW (Figure 7B).

### 3.8 Effects of MESL on tumor weight gain and survival time in EAC cell bearing mice

The effects of MESL on tumor weight gain and mean survival time (MST) at different doses have been summarized in Table 2. It has been observed that the life span of EAC cell-bearing mice treated with MESL was increased by 28.45% and 48.52% at doses 50 and 100 mg/kg BW compared to control mice, respectively, which was comparable to bleomycin (80.35%). It was also found that the percentage of the increased life span of EAC cell-bearing mice treated with MESL at the dose of 100 mg/kg BW is more than the percentage of increased life span in MESL treated mice at the dose of 50 mg/kg BW. In control (EAC bearing) group, the tumor weight was increased by 14.2 gm on 20 days compared to the control mice. The tumor weight was significantly decreased in mice treated with MESL at doses 50 mg/kg BW and 100 mg/kg BW, compared to that of control mice.

**Table 2:** Effects of MESL on survival time and BW gain of EAC cell-bearing mice.

Mice groups	MST (day)	%ILS	BW gain (gm) after 20 days of EAC cells inoculation
EAC + control	22.4 ± 1.14	-	14.2 ± 2.94
EAC + MESL (50 mg/kg)	29.2 ± 2.16∗	28.45 ± 7.23∗	7.21 ± 1.78∗
EAC + MESL (100 mg/kg)	33.53 ± 1.30∗	48.52 ± 9.53∗	5.8 ± 1.48∗
EAC + Bleomycin (0.3 mg/kg)	40.4 ± 2.4∗	80.35 ± 10.75∗	4.2 ± 1.30∗

All data were expressed as mean ± SD (n = 6); ∗*p* < 0.05 compared to EAC control; MST: survival time; %ILS: percentage increase of life span.

### 3.9 Effects of MESL on morphological change of EAC cells

**Figure 8:**
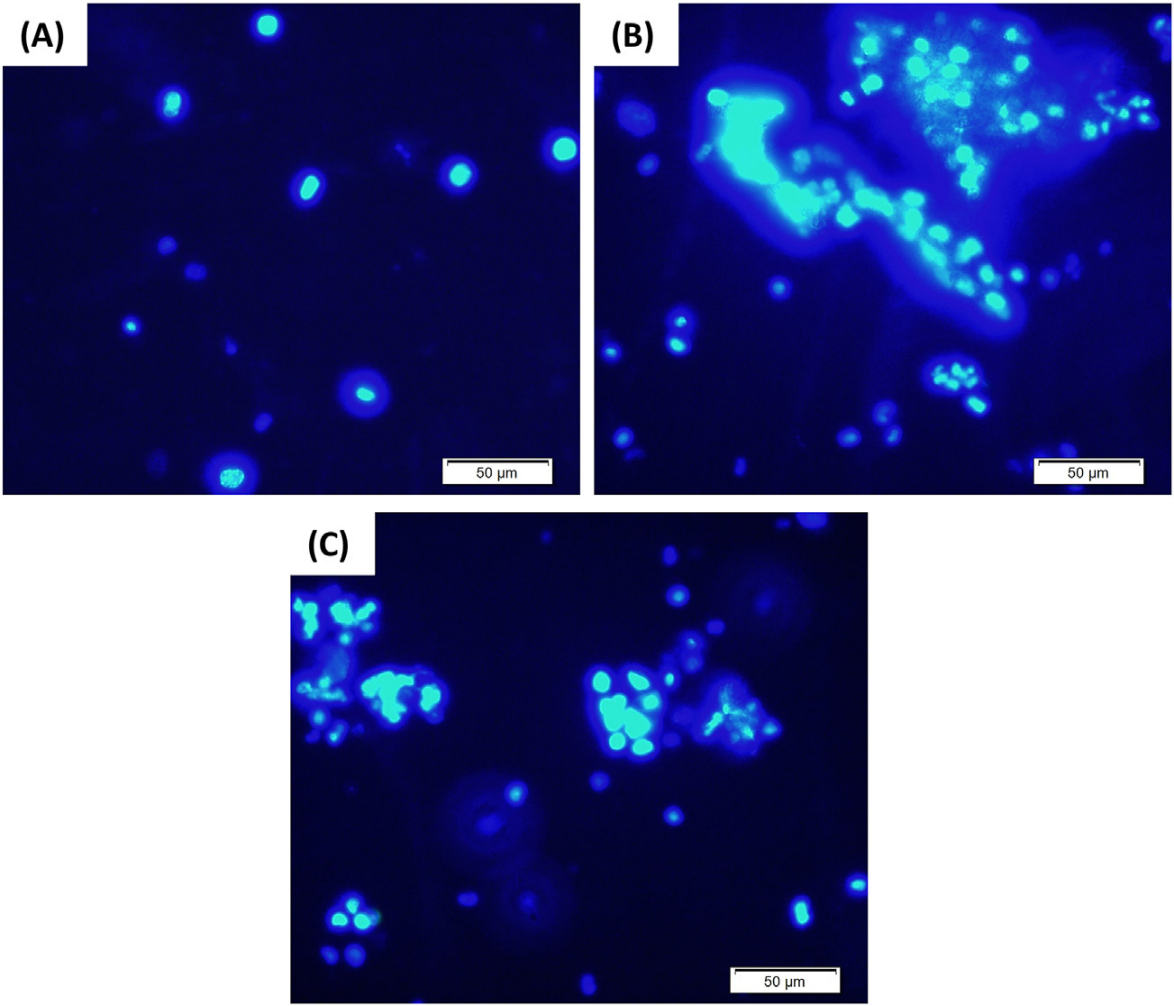
Fluorescence microscopic view of EAC cells collected from all three groups of EAC cell-bearing mice. Fig (A), (B), and (C) represent Hoechst 33342 stained EAC cells in control mice, and MESL treated mice at the dose of 50 mg/kg BW and100 mg/kg BW, respectively.

The effects of MESL treatment on the apoptotic death of EAC cells were also noted in this study. In EAC cell-bearing control mice, apoptotic features such as nuclear condensation and fragmentation, cell membrane blebbing, apoptotic bodies, loss of contact with neighboring cells were not found (Figure 8A). However, MESL treatment showed increased apoptosis of EAC cells in a dose-dependent manner (Figure 8B and C).

### 3.10 Effects of MESL on hematological parameters

Hematological parameters were also found to be altered from typical values along with the growth of tumors in EAC cell-bearing mice. Hgb, RBC, and WBC count were decreased after inoculation of EAC cells in Swiss albino mice (Table 3). After treatment with MESL, it was found that treated mice's hematological parameters were restored moderately at both doses (Table 3).

**Table 3:** Effects of MESL on hematological parameters of EAC cell bearing mice.

Parameters	Normal group	EAC + control	EAC+50 mg/kg BW	EAC+100 mg/kg BW	Bleomycin 0.03 mg/kg BW
Hgb (gm/dL)	16.46 ± 0.58	9.54 ± 0.28	11.864 ± 0.56∗	13.43 ± 0.67∗	14.18 ± 0.36∗
RBC(x10^9^)cells/mL)	7.52 ± 0.46	2.52 ± 0.30∗	3.61 ± 0.36∗∗	6.24 ± 00.25∗	7.12 ± 0.14∗
WBC(x10^6^)cells/mL)	8.20 ± 1.92	44.40 ± 2.96∗	24.6 ± 1.81∗∗	12.8 ± 0.83∗	10.34 ± 0.26∗

All data were expressed as mean ± SD (n = 6); ∗*p* < 0.05 compared to EAC cells bearing control mice; ∗∗*p* < 0.05 compared to normal control mice.

## 4 Discussion

All data of this study showed that MESL contains higher amounts of polyphenols and possesses antioxidant and free radical reducing capacity. MESL also showed significant inhibition of the EAC cell proliferation, tumor growth, and increased apoptotic death of EAC cells accompanied by restoring hematological parameters in EAC cell-bearing mice. Till now, there is no data of MESL against EAC cell.

EAC is one of the most common experimental tumor models used globally. EAC is highly preferable because of its rapid proliferation, high transplantability, shorter life span, and 100% malignancy [30]. Nowadays, cancer treatments are well developed through chemotherapy, radiotherapy, or hormone therapy. However, all of them have adverse side effects, and it is a great challenge to treat cancer without side effects [31]. Therefore researchers focus on biologically active plant-derived secondary metabolites such as flavonoids, polyphenols, alkaloids, and steroids for cancer treatment [32]. From various studies, it was found that plant-derived components have antineoplastic role against EAC infected mice and increase mean survival time and life span [28, 33, 34, 35].

The consumption of a diet rich in beneficial plant metabolites can lower the risk of several diseases in humans, including different types of cancer [36]. Plant-derived foods contain bioactive compounds including vitamins, polyunsaturated fatty acids, and phytochemicals having therapeutic potentiality [37, 38]. Naturally occurring phytochemicals possess prominent biological activities such as anti-inflammatory, antioxidant, antimicrobial, antidiabetic, and anticancer properties [12, 37]. With other bioactive compounds present in the plant, the nutritional value and medicinal properties of plant-derived foods largely depend on their overall flavonoid and phenolic contents [39]. In this study, we found higher amounts of phenolics and flavonoids present in MESL, and this finding is consistent with previous reports [40]. These polyphenols present in MESL may be associated with its antioxidant activity.

To confirm that, we have explored the antioxidant property of MESL by using five different assays. Among those assays, DPPH, ABTS, and NO are known as scavenging assays, whereas FRAP and CUPRAC are considered ion-reducing assays. Reactive oxygen species (ROSs) in a biological system are constantly created and eliminated in the living organism. They play essential roles in various normal biochemical functions and abnormal pathological processes [41]. They are produced in all aerobic organisms during metabolism and play an essential role in cancer, although a low level of ROSs exhibit beneficial roles [42]. Antioxidants are highly reactive to ROSs and have the potentiality to reduce or scavenge free radicals produced during oxidative stress. The ability experimental sample to suppress radical cation can be evaluated by FRAP and CUPRAC assays [43]. The FRAP assay for determining antioxidant capacity is very common, whereas the CUPRAC assay is a relatively new assay in which neocuproine is used as a chromogenic oxidizing agent. In both FRAP and CUPRAC assays, MESL showed potent antioxidant capacity in this study. DPPH and ABTS are mostly typical scavenging assays to determine the free radical scavenging capacity. In DPPH and ABTS assays, antioxidant molecules quench the free radicals and decrease the intensity of color by donating an electron and providing hydrogen atoms [44, 45]. In this study, MESL showed potent scavenging activity. At the dose of 100 μg/mL, MESL scavenged 89.63% ABTS radical and 79.43% DPPH free radical. Another standard assay widely used to determine the scavenging capacity of the experimental sample is the NO scavenging assay [46]. In this assay, MESL showed IC_50_ value of 70.50 μg/mL. Therefore, *S. calendulacea* leaf might be helpful for the treatment of diseases associated with oxidative damage, such as neurodegenerative diseases, diabetes, and cancer [12, 37].

The positive correlation between oxidative stress and cancer is well known nowadays. Oxidative stress is associated with both cancer induction and progression [47, 48]. Therefore, molecules having antioxidant properties can be helpful for the treatment and prevention of cancers [12]. In this study, we have also explored the anticancer activity of MESL against EAC cell-bearing Swiss albino mice. In our present study, MESL showed significant inhibition in the growth of EAC cells and tumor weight in EAC cell-treated mice. MESL also increased the apoptotic death of tumor cells and increased the survival time of EAC cell-bearing mice. Morphologically, MESL treated EAC cells had apoptosis characteristics such as membrane blebbing or apoptotic bodies, while the untreated control EAC cells had regular and circular form showed in Figure 8. Additionally, MESL treatment restored altered hematological parameters such as Hgb, WBC, and RBC in tumor-bearing mice in this study. All findings of this study suggest that the MESL may have antineoplastic characteristics because its phytoconstituents have antioxidative properties.

## 5 Conclusion

The present study revealed that *S. calendulacea* leaves are a natural source of different bioactive phytochemicals having potent antioxidant activity. It has the potentiality to inhibit EAC cell proliferation, decrease tumor weight, increase the survival time of EAC cell-bearing mice and promote apoptotic death of EAC cells. These data indicate the antioxidant and antineoplastic properties of *S. calendulacea* leaves. However, further study is needed to explore specific bioactive compounds, their structures, and their mechanism of action present in *S. calendulacea* leaves associated with its antineoplastic property.

## Declarations

### Author contribution statement

Md. Wasim Baria: Conceived and designed the experiments; Performed the experiments; Analyzed and interpreted the data; Wrote the paper.

Ariful Islamb: Analyzed and interpreted the data; Contributed reagents, materials, analysis tools or data; Wrote the paper.

Md. Monirul Islamc, Md. Mahbubur Rahman Khand: Performed the experiments; Analyzed and interpreted the data.

Mst Julia Sultanaa: Performed the experiments; Analyzed and interpreted the data; Wrote the paper.

Rashida Afroza: Analyzed and interpreted the data; Wrote the paper.

Salina Shaheen Parule: Analyzed and interpreted the data; Contributed reagents, materials, analysis tools or data.

A. M. Swarazf: Performed the experiments; Contributed reagents, materials, analysis tools or data; Wrote the paper.

Mohammad Ismail Hossaina: Conceived and designed the experiments; Contributed reagents, materials, analysis tools or data; Wrote the paper.

Mohammad Amirul Islam: Conceived and designed the experiments; Analyzed and interpreted the data; Contributed reagents, materials, analysis tools or data; Wrote the paper.

### Funding statement

This research did not receive any specific grant from funding agencies in the public, commercial, or not-for-profit sectors.

### Data availability statement

Data will be made available on request.

### Declaration of interests statement

The authors declare no conflict of interest.

### Additional information

No additional information is available for this paper.
